# Measuring the Environmental Efficiency and Technology Gap of PM_2.5_ in China’s Ten City Groups: An Empirical Analysis Using the EBM Meta-Frontier Model

**DOI:** 10.3390/ijerph16040675

**Published:** 2019-02-25

**Authors:** Shixiong Cheng, Jiahui Xie, De Xiao, Yun Zhang

**Affiliations:** 1 School of Business, Hubei University, Wuhan 430062, China; csxcsx007@hubu.edu.cn (S.C.); jiahui0821@stu.hubu.edu.cn (J.X.); xiaode@hubu.edu.cn (D.X.); 2 Institute for Open Economy Research Centre, Hubei University, Wuhan 430062, China; 3 School of Finance, Shanghai Lixin University of Accounting and Finance, Shanghi 201620, China

**Keywords:** PM_2.5_, epsilon-based measure model, meta-frontier Malmquist index, city group, directional distance function

## Abstract

Since air pollution is an important factor hindering China’s economic development, China has passed a series of bills to control air pollution. However, we still lack an understanding of the status of environmental efficiency in regard to air pollution, especially PM_2.5_ (diameter of fine particulate matter less than 2.5 μm) pollution. Using panel data on ten major Chinese city groups from 2004 to 2016, we first estimate the environmental efficiency of PM_2.5_ by epsilon-based measure (EBM) meta-frontier model. The results show that there are large differences in PM_2.5_ environmental efficiency between cities and city groups. The cities with the highest environmental efficiency are the most economically developed cities and the city group with the highest environmental efficiency is mainly the eastern city group. Then, we use the meta-frontier Malmquist EBM model to measure the meta-frontier Malmquist total factor productivity index (MMPI) in each city group. The results indicate that, overall, China’s environmental total factor productivity declined by 3.68% and 3.49% when considering or not the influence of outside sources, respectively. Finally, we decompose the MMPI into four indexes, namely, the efficiency change (EC) index, the best practice gap change (BPC) index, the pure technological catch-up (PTCU) index, and the frontier catch-up (FCU) index. We find that the trend of the MMPI is consistent with those of the BPC and PTCU indexes, which indicates that the innovation effect of the BPC and PTCU indexes are the main driving forces for productivity growth. The EC and FCU effect are the main forces hindering productivity growth.

## 1. Introduction

China’s air pollution has seriously affected the country’s international image and social development [1]. Air pollution, especially PM_2.5_ pollution (diameter of fine particulate matter less than 2.5 μm), also threatens the health of people in China. According to Chen et al. [2], in China, statistics show that the total particulate matter pollution has increased by 100 μg/m^3^, leading to a reduction in average life expectancy of 3 years. Since 2012, central government policymakers in Beijing have also recognized the seriousness of haze pollution and formulated a series of measures to control haze pollution. For example, China has proposed a new Ambient Air Quality Standard (GB3095-2012) and issued a new Air Pollution Prevention and Control Action Plan to reduce heavy pollution weather through five years of hard work. In addition, China is also facing the pressure of economic transformation. China’s past high energy consumption-high pollution-high growth model is no longer sustainable. However, haze pollution is an obstacle to high-quality economic growth in China [3]. As the world’s second-largest economy, sustainable economic growth in China is also very necessary [4]. The question of how to control haze pollution without hindering economic growth is important. Measuring and assessing the environmental efficiency of China in recent decades under haze pollution would be very useful [5].

In the nearly 40 years since the reform and the opening up of China to international trade, China’s urbanization process has accelerated, accompanied by the rapid development of China’s economy. Some cities in China have formed city groups through regional linkages and coordinated development. However, from the perspective of the distribution of heavily hazed polluted areas, China’s haze pollution has a typical regional distribution and pollution aggregation characteristic. The haze pollution in key city groups, such as the Beijing-Tianjin-Hebei (BTH), Yangtze River Delta (YRD), and Pearl River Delta (PRD) city groups and ChengYu, has always been very serious compared to other regions. Therefore, studying haze pollution from the perspective of city groups would be very meaningful.

To properly assess environmental efficiency and productivity in China from the perspectives of PM_2.5_ pollution and city groups, this paper uses panel data on PM_2.5_ pollution in China’s ten major city groups, including 100 prefecture-level cities and municipalities. Based on our existing findings, this study is the first to research Chinese PM_2.5_ pollution environmental efficiency from a regional perspective. For a better measurement of environmental technology efficiency and productivity in regard to the city groups, the meta-frontier model is very suitable. This paper is also the first to use the epsilon-based measure (EBM) meta-frontier model. In addition, PM_2.5_ pollution is different from general pollutants such as CO_2_ and SO_2_. The PM_2.5_ concentration not only is produced by local areas but will spread across regions [6]. Therefore, measuring the PM_2.5_ concentration and PM_2.5_ efficiency from a cross-regional perspective would also be very meaningful. To accurately measure environmental efficiency under PM_2.5_ pollution, this paper is the first study to consider the impact of external pollution sources on PM_2.5_ pollution.

Following this introduction, the rest of the paper is structured as follows: Section 2 is the literature review; Section 3 introduces the EBM meta-frontier model; Section 4 describes the data source and descriptive statistics; Section 5 presents the empirical results, and the last section is the conclusion and policy implications.

## 2. Literature Review

Multiple input–output efficiency evaluation is mainly based on parametric and non-parametric methods. The parametric methods are divided into index methods and stochastic frontier analysis (SFA) methods. Both the index methods and the SFA methods need to set a specific production function form. The difference between the two methods is that the index methods must artificially set parameter values, and the SFA methods must estimate the parameter values based on an econometric method. In contrast, non-parametric methods based on data envelopment analysis (DEA) do not need to set specific function forms, nor do they need to estimate specific parameter values. Thus, the use of DEA methods is more convincing. An increasing number of scholars have used DEA methods to conduct energy and environmental efficiency (E&E) studies. Zhou et al. [7] reviewed more than 100 environmental efficiency studies using the DEA method. More recently, Sueyoshi et al. [8] provided a survey of 693 E&E studies using the DEA methodology from the 1980s to 2010s. The number of articles on DEA applications for energy and the environment has increased dramatically, especially since the 2000s. According to Sueyoshi, Yuan and Goto [8], previous research focused on energy electricity, oil, coal, gas, heat, renewables, energy efficiency or energy saving, the environment or sustainability.

Traditional DEA models, do not consider the effect of an undesirable output on the desirable output, which is unsuitable for practical production. Based on traditional DEA models, an extended directional distance function (DDF) model was proposed by Chung et al. [9]. The major advantage of this DDF model is that it can simultaneously expand the desirable output and reduce the undesirable output and exploit the weak disposability of the undesirable output [10]. The DDF model can measure E&E more accurately than traditional DEA models [11,12]. Therefore, an increasing number of E&E studies have used the DDF model to evaluate Chinese environmental efficiency in different areas. For example, Watanabe and Tanaka [13] used the DDF model to measure the environmental efficiency of Chinese industry at the provincial level from 1994 to 2002. A similar study can also be found in Yuan et al.’s [14] research on Chinese industry. Wang et al. [15] used a multi-DDF model to measure Chinese regional energy and emissions efficiency. There are also studies using the DDF model for a certain industry. He et al. [16] used a DDF model to measure energy efficiency and productivity change in China’s iron and steel industry. Zhang and Choi [17] evaluated fossil fuel power plants for the 2005 to 2010 period in China. For more papers in regard to a literature review, see Zhang and Choi [10].

However, the traditional DEA models, such as the DEA–BCC [18] model and the DEA–CCR model [19], are all radial models, which means that they calculate efficiency by measuring an inefficiency score that represents the projection distance from the observed decision-making units (DMUs) to the efficiency frontier according to Sueyoshi and Goto [20]. In addition, traditional radial DDF models require the input variables, desirable output variables, and undesirable output variables to vary by the same proportion. However, the radial models cannot avoid efficiency overestimation due to the existing slack variables of the output or input. In contrast, as the slacks represent inefficiency, non-radial models can evaluate overall efficiency by measuring a total amount of slack. Tone [21,22] proposed a slack-based measure (SBM) model that enters the slack variable directly into the objective function and solves the problem of efficiency evaluation under the input slack and output slack, as well as avoiding the influence of radial and oriented choice. There are many studies using SBM models to evaluate E&E in China from different perspectives. Zhou et al. [23] measured the environmental efficiency of industrial sectors in China by an improved SBM model. Li et al. [24] utilized a super-efficiency SBM model to evaluate China’s regional E&E. Zhang and Choi [25], who measured China’s regional economies from 2001 to 2010, performed a similar study. Xiao et al. (2018) used a super-efficiency SBM model with undesirable outputs (S-U-SBM) to evaluate the E&E of 31 sectors in China. Zhang et al. [26] used the duality theory of SBM models to measure the environmental efficiency and shadow price of the Poyang Lake Ecological Economics Zone in China. Deng et al. [27] used the SBM model to estimate the water use efficiency of 31 provinces in China in 2004 to 2013.

However, although SBM models consider the existence of slacks, they do not assume a proportional adjustment of inputs and outputs; they assume that the non-radial slack change is too loose to lose the original information on inputs and outputs. In the linear programming solution process, the SBM model is exposed as insufficient; that is, the optimal solution of the zero value and the positive value are significantly different. Most importantly, the SBM model has some difficulty in understanding economic meaning. Therefore, it is necessary to combine the radial model and the non-radial model into a composite model to obtain a more accurate measure of efficiency. Thus, a hybrid distance model, namely, the EBM model, was first proposed by Tone and Tsutsui [28]. The EBM model combines the advantages of radial and non-radial measures into a hybrid linear programming framework, and it can better overcome the disadvantages of radial and non-radial models. In recent years, an increasing number of articles have used it to measure energy efficiency. For example, a global EBM model was used by Qin et al. [29] to measure the energy efficiency in China’s coastal regions. Cui and Li [30] used dynamic EBM models to measure the efficiency of 19 airlines. Xu and Cui [31] also proposed a new network EBM model to evaluate airline energy efficiency. Therefore, due to the advantages of the EBM model in measuring efficiency, this paper attempts to use the EBM model to obtain a more accurate environmental efficiency evaluation under PM_2.5_ constrictions. To the best of my knowledge, this study is the first attempt to use the EBM model to evaluate PM_2.5_ efficiency. This study can provide some new evidence and strategies for this kind of problem.

Both the EBM model and SBM model are based on cross-sectional data and are used to measure the environmental efficiency of the DMU in a certain period. However, in this case, efficiency cannot be compared across periods. The Malmquist productivity index (MPI), proposed by Caves et al. [32], can be used for inter-temporal comparisons. Fare et al. [33] extended the MPI and decomposed it into an efficiency change (EC) index and a technological change (TC) index. However, the MPI does not consider the existence of undesirable outputs. Therefore, Färe et al. [34] and Chung, Färe and Grosskopf [9] formulated a Malmquist–Luenberger productivity index (MLPI) that can compare environmental efficiencies under an undesirable output. Nonetheless, in the above models, such as the MPI or MLPI, the DMUs are usually considered as an element of the same production set with the same technology. However, in fact, DMUs tend to be technologically heterogeneous. Therefore, according to Oh [35], the production efficiency of different production sets cannot be directly compared. Thus, Oh and Lee [36] first proposed a meta-frontier Malmquist model and decomposed it into three different indexes, namely, the EC index, the best practice gap change (BPC) index and the technology gap change (TGC) index. Chen and Yang [37] extended the meta-frontier Malmquist productivity and decompose the catch-up index into the pure technological catch-up (PTCU) index and the frontier catch-up (FCU) index. Because the meta-frontier model can takes into account technological heterogeneity, an increasing number of studies use this model to measure environmental efficiency. Zhang and Choi [17] used the meta-frontier non-radial Malmquist index (MNMCPI) to measure total factor CO_2_ emissions performance in China. Munisamy and Arabi [38] incorporated an SBM index and meta-frontier MLPI to evaluate the productivity change in 48 Iranian thermal power plants. A new meta-frontier Malmquist index was adopted by Yao et al. [39] to estimate China’s CO_2_ emissions performance and to decompose the index into three indexes to analyse its driving force. Feng et al. [40] utilized a three-hierarchy meta-frontier data envelopment method to evaluate the energy efficiency and energy-saving potential of China. There are also many studies based on a certain sector or industry. For example, Long et al. [41] evaluated the environmental efficiency of the YRD’s 192 thermal power plants. Li et al. [42] measured the environmental efficiency of fossil fuel power plants across provinces using meta-frontier Malmquist indexes. A similar study with power plants can also be found in Wang et al. [43]. Fei and Lin [44] measured China’s agricultural efficiency by applying the meta-frontier framework. Feng et al. [45] and Tian and Lin [46] performed similar research on China’s metal industry and light industry sectors.

According to previous studies, we find that there is scarce research that focuses on air pollution and environmental efficiency, to say nothing of Chinese air pollution. There are only a few papers focused on this area. For example, Sueyoshi and Yuan [47] used a radial DEA model to measure the efficiency of cities in China, with PM_2.5_ and PM_10_ as undesirable outputs. Xu et al. [48] examined the key driving forces of PM_2.5_ emissions in China by a non-parametric additive regression model. Wei et al. [49] also investigated the key sectors contributing to air pollutant emissions and test the characteristics of the regional heterogeneity of air pollution in China. Zhou et al. [50] measured the air quality of 31 main cities using a zero-sum gain DEA approach and daily air quality index (AQI) data.

The position of this study: Motivated by the proposed research gaps, this paper will evaluate environmental efficiency under PM_2.5_ constrictions by considering the technology heterogeneity in different city groups. The research in this paper can be summarized based on the following innovations, all of which are found by comparing the aforementioned research summarized above. First, few studies have considered the influence of PM_2.5_ as an undesirable output for E&E studies. In cases in which air pollution has become very serious, the previous research on China’s environmental problems is clearly inconsistent with China’s reality. Currently, people in Chinese have long suffered from air pollution. Therefore, the practical significance of previous research is insufficient to discuss Chinese environmental issues. The proposed study is the first research effort to incorporate the most serious undesirable outputs that are the by-products of Chinese economic growth. Second, unlike previous studies on air pollution efficiency measurement, this study is the first to analyse the impact of outside sources on internal PM_2.5_. Third, city groups are used to analyse PM_2.5_ efficiency because the diffusion of PM_2.5_ and Chinese air pollution always have regional characteristics. It is very important to analyse PM_2.5_ in city groups, and unlike most efficiency gap studies, we use city groups to classify the group frontier. Finally, a new meta-frontier DEA EBM model that can model radial and non-radial measures properly and that exhibits the weak disposability of the desirable and undesirable outputs and can be used for a group frontier and a meta-frontier is proposed. To the best of my knowledge, this study is the first research to use this model to analyse air pollution in China.

## 3. Model

We assume that there are K DMUs. Each DMU uses M input vectors $x=\left(x_{1k,}x_{2k,}\ldots ,x_{Mk}\right)$ to jointly produce q_1_ desirable output vectors $y=\left(y_{1k,}y_{2k,}\ldots ,y_{q_{1}k}\right)$ and q_2_ undesirable output vectors $b=\left(b_{1k,}b_{2k,}\ldots ,b_{q_{2}k}\right)$. According to Chambers et al., (1996), multi-output production technology can be defined as follows:

(1) P(x)={(y,b):x       can     prodcue     (y,b)}

To exclude the loss of generality, production technologies must also satisfy the following properties:

(1) P(0)=(0,0).
(2) if (y,b)∈P(x) and b=0, then y=0.
(3) if (y,b)∈P(x), then for 0<θ<1, (θy,θb)∈P(x).
(4) if (y,b)∈P(x), then for $y^{\prime }\leq y$, $\left(y^{\prime },b\right)\in P(x)$.
(5) if $x^{\prime }\geq x$, then $P(x)\subseteq P(x^{\prime })$.

The first and second properties represent the null jointness of the desirable and undesirable outputs, respectively; that is, the bad outputs are not avoidable during production. The third property represents weak disposability (i.e., any proportional decrease in satisfying and unsatisfying outputs simultaneously is feasible). The fourth property represents a desirable output that is strongly disposable, which implies that the directional output distance function is monotonic in the desirable outputs. The fifth property represents the inputs that are freely disposable.

Similar to Färe et al. [51], the production set (S) satisfying with all of the above properties as well as the constant returns to scale (CRS) assumption can be expressed as follows:

(2) S={(y,b) |x≥Xλ,y≤Yλ,b=Bλ,λ≥0}

where λ is an intensity vector for weighting each element. *X*; *Y*, and *B* represent the matrix of input index, desirable outputs index, and undesirable outputs index, respectively.

The DDF is a generalized form of the Shephard distance function, which was first proposed by and developed by Chung, Färe, and Grosskopf [9]. According to Chung, Färe, and Grosskopf [9], the input-oriented DDF model under the CRS (constant returns to scale) assumption can be defined as follows:

(3) $$\min\;\theta \;s\;s.t.\;\begin{array}{l} X\lambda \leq \theta x\\ Y\lambda \geq y\\ B\lambda =b\\ \lambda \geq 0 \end{array}$$

where λ represents the Lagrange multiplier vector and θ denotes the input-oriented efficiency measure. We can also give the ∑λ=1 constraint for the DDF model to meet the assumption of the variable returns to scale (VRS) conditions.

Similar to Equation (3), the output-oriented DDF model can be defined as follows:

(4) $$D_{0}(x,y,b;g)=\max\;\varphi s\;s.t.\;\begin{array}{l} X\lambda \leq x\\ Y\lambda -\varphi y\geq y\\ B\lambda +\varphi b\leq b\\ \lambda \geq 0 \end{array}$$

where φ denotes the output-oriented DDF efficiency measure.

According to Tone [22], to better address the existence of input excesses and output shortfalls as well as the slacks of DMUs, the SBM model is suitable for efficiency evaluation. Thus, based on the assumption of CRS and weak disposability, the SBM model is formulated as follows:

(5) $$\min\;\rho =\frac{1-\frac{1}{m}\sum_{i=1}^{m}s_{i}^{-}/x_{ik}}{1+\frac{1}{q_{1}+q_{2}}\left(\sum_{r=1}^{q_{1}}s_{r}^{+}/y_{rk}+\sum_{n=1}^{q_{2}}s_{n}^{b-}/b_{nk}\right)}\;s.t.\;\begin{array}{l} X\lambda +s^{-}=x_{k}\\ Y\lambda -s^{+}=y_{k}\\ B\lambda +s^{s.t.\;b-}=b_{k}\\ s^{-}\geq 0,s^{+}\geq 0,s^{b-}\geq 0,\lambda \geq 0 \end{array}$$

where $s_{i}^{-}$, $s_{r}^{+}$, and $s_{n}^{b-}$ are slack variables that represent the input excess, desirable output shortfall, and undesirable output excess, respectively.$s^{-}$, $s^{+}$, and $s^{b-}$ indicate the vector form of the $s_{i}^{-}$, $s_{r}^{+}$, and $s_{n}^{b-}$ variables. As mentioned above, *m* represents the number of inputs, *q_1_* represents the number of desired outputs, *q*_2_ represents the number of undesired outputs. The objective function value (ρ) is the efficiency estimator, which ranges from zero to one.

As we can see from the previous DDF and SBM models, the traditional DDF model always represents a radial measure, and the SBM model represents a non-radial measure. The traditional DDF model mainly deals with a proportionate reduction in input or output resources. In contrast, the non-radial SBM model can obtain the maximum rate of reduction in inputs and allows independent changes to the associated slacks. The main shortcoming of the traditional DDF model is that it neglects non-radial slacks in evaluating the efficiency score, while the projected DMU in the SBM model may lose proportionality in the original inputs because the slacks are not necessarily proportional to the inputs; thus, it is difficult to explain the economic implications of the SBM efficiency estimator. To integrate the advantages and deal with the disadvantages of radial and non-radial models, Tone and Tsutsui [28] developed a hybrid distance composite model, the EBM, which combines both radial and non-radial features in a unified framework.

The no-oriented EBM model under the CRS assumption can be combined with the Equations (4) and (5), and expressed as follows:

(6) $$\min\;\rho =\frac{\theta -\varepsilon^{-}\frac{1}{\sum_{i=1}^{m}\omega_{i}^{-}}\sum_{i=1}^{m}\omega_{i}^{-}s_{i}^{-}/x_{ik}}{\varphi +\varepsilon^{+}\times \frac{1}{2}\left(\frac{1}{\sum_{r=1}^{q_{1}}\omega_{r}^{+}}\sum_{r=1}^{q_{1}}\omega_{r}^{+}s_{r}^{+}/y_{rk}+\frac{1}{\sum_{n=1}^{q_{2}}\omega_{b}^{-}}\sum_{n=1}^{q_{2}}\omega_{b}^{-}s_{t}^{b-}/b_{nk}\right)}\;s.t.\;\begin{array}{l} X\lambda +s^{-}=\theta x_{k}\\ Y\lambda -s^{+}=\varphi y_{k}\\ B\lambda +s^{s.t.\;b-}=\varphi b_{k}\\ s^{-}\geq 0,s^{+}\geq 0,s^{b-}\geq 0,\lambda \geq 0\\ \theta \leq 1,\varphi \geq 1 \end{array}$$

where $\omega_{i}^{-}$, $\omega_{r}^{+}$, and $\omega_{b}^{-}$ denote the weights of the *i*th input, the *r*th good output, and the nth bad output and satisfy $\sum_{i=1}^{m}\omega_{i}^{-}=1$, $\sum_{r=1}^{q_{1}}\omega_{r}^{+}=1$, and $\sum_{n=1}^{q_{2}}\omega_{b}^{-}=1$. The weights are the relative importance of each input, good output or bad output measurement. $\varepsilon^{-}$ and $\varepsilon^{+}$ are key parameters.$\varepsilon^{-}$ indicates the relative importance of the non-radial slacks over the radial θ, and $\varepsilon^{+}$ indicates the relative importance of the non-radial slacks over the radial φ; the range of $\varepsilon^{-}$ and $\varepsilon^{+}$ is [0,1], which is equivalent to the radial model when $\varepsilon^{-}$ and $\varepsilon^{+}$ are equal to zero and to the non-radial model when $\varepsilon^{-}$ and $\varepsilon^{+}$ are equal to one.

### Measure of the Group Technology Gap

The EBM model is a static measurement. To discover the dynamic trend of environmental efficiency, the Malmquist–Luenberger meta-frontier approach model was introduced. According to O’Donnell et al. [52], DMUs with the same technology are categorized as the same group, and all DMUs can be divided into J groups with different technologies.

To begin with the definition and decomposition of the meta-frontier MPI, three different technology sets should be defined. Suppose that there are *t* = 1, …, *T* periods: a reference production set at each point is named the contemporaneous benchmark technology set, according to Pastor and Lovell [53], for group j in period t as $P_{t}^{j}=\left\{\left(y_{t}^{j},b_{t}^{j}\right):\;x_{t}^{j}\;can\;prodcue\;\left(y_{t}^{j},b_{t}^{j}\right)\right\}$. According to Oh and Lee [36], an inter-temporal benchmark technology set I is defined as $P_{j}^{I}=\left\{P_{j}^{1}\cup P_{j}^{2}\cup \ldots \cup P_{j}^{T}\right\},j=1\ldots J$, and a global benchmark technology set G is defined as $P^{G}=\left\{P_{1}^{I}\cup P_{2}^{I}\cup \ldots \cup P_{J}^{I}\right\}$. According to O’Donnell, Rao and Battese [52], combining with Equation (4), the technology gap ratio can be defined as follows:

(7) $$0\leq TGR(.^{t})=\frac{D^{G}(.^{t})}{D^{I}(.^{t})}=\frac{TE^{I}}{TE^{G}}\leq 1$$

Equation (7) indicates that the meta-frontier is an envelopment of the group frontiers. $D^{G}(.^{t})$ and $D^{I}(.^{t})$ represent the DDF measure under the conditions of the global frontiers and meta-frontier measure in period *t*. Because the decomposition in this section requires many notations, we replace all distance function $D(x^{t},y^{t},b^{t})$ as $D(.^{t})$ to save space. *TE^I^* is the technological efficiency measure based on the group frontiers, and *TE^G^* is the technological efficiency measure based on the meta-frontier. *TGR* is the technology gap ratio, which represents the ratio between the group and global technology; it ranges from [0,1]. The closer the TGR is to one, the closer the group technology is to the global technology. The value of the *TGR* tends to zero, which means that the group technology is farther away from the global technology.

As developed by Caves, Christensen and Diewert [32], a contemporaneous Malmquist productivity index (MPI) measuring the productivity change between periods *t* and *t* + 1, is defined as:

(8) $$MPI(.^{t},.^{t+1})={\left[\frac{D^{t}\left(.^{t+1}\right)}{D^{t}\left(.^{t}\right)}\times \frac{D^{t+1}\left(.^{t+1}\right)}{D^{t+1}\left(.^{t}\right)}\right]}^{\frac{1}{2}}$$

where $D^{t}\left(.^{t+1}\right)$ represents the DDF measure in *t* periods, DMUs based on the *t* + 1 periods technology, and other distance function variables have the same meanings.

Here, we define the MMPI (the meta-frontier Malmquist total factor prouctivity index) based on the global technology set (G) as follows:

(9) $$MMPI=M^{G}(.^{t},.^{t+1})=\frac{D^{G}(.^{t+1})}{D^{G}(.^{t})}$$

Similar to Oh (2010), we can decompose the MMPI as follows:

(10) $$\begin{array}{l} MMPI=\frac{D^{G}(.^{t+1})}{D^{G}(.^{t})}=\frac{D^{t+1}(.^{t+1})}{D^{t}(.^{t})}\times \left\{\frac{D^{t}(.^{t})}{D^{t+1}(.^{t+1})}\times \frac{D^{G}(.^{t+1})}{D^{G}(.^{t})}\right\}\\ =\frac{D^{t+1}(.^{t+1})}{D^{t}(.^{t})}\times \left\{\frac{D^{t}(.^{t})}{D^{t+1}(.^{t+1})}\times \frac{D^{I}(.^{t+1})}{D^{I}(.^{t})}\right\}\times \left\{\frac{D^{I}(.^{t})}{D^{I}(.^{t+1})}\times \frac{D^{G}(.^{t+1})}{D^{G}(.^{t})}\right\}\\ =\frac{TE^{t+1}}{TE^{t}}\times \frac{BPG^{I,t+1}}{BPG^{I,t}}\times \frac{TGR^{t+1}}{TGR^{t}}\\ =EC\times BPC\times TGRC \end{array}$$

where EC (the efficiency change index) denotes the efficiency change, and BPC (the best practice gap change index) stands for the best practice gap change, which measures the technology gap between the current technological frontier and the inter-temporal period technological frontier. Therefore, it also represents the effect of technological progress or technological degradation. The BPC index equals one if and only if the particular DMU is located on the inter-temporal frontier. TGRC (the technology gap ratio change index) stands for the technological gap change, which represents the change in technology leadership. Due to technological changes, the technology gap between the group and global technology will also change.

Similar to Chen and Yang [37], the TGRC index can be further decomposed as follows:

(11) $$\begin{array}{l} TGRC={\left[\frac{TGR^{t+1}(.^{t+1})}{TGR^{t}(.^{t})}\times \frac{TGR^{t}(.^{t+1})}{TGR^{t+1}(.^{t})}\right]}^{\frac{1}{2}}\\ =\frac{TGR^{t+1}(.^{t+1})}{TGR^{t}(.^{t})}\times {\left[\frac{TGR^{t}(.^{t+1})}{TGR^{t+1}(.^{t+1})}\times \frac{TGR^{t}(.^{t})}{TGR^{t+1}(.^{t})}\right]}^{\frac{1}{2}}\\ =PTCU\times FCU \end{array}$$

where PTCU (the pure technological catch-up index) denotes pure technological catch-up because it purely captures the catch-up in technology without the elements of technological inefficiency from the view of a group frontier; FCU (the frontier catch-up index) denotes frontier catch-up because the FCU index captures the velocity of change of the meta-frontier relative to that of the group frontier.

Combined with Equations (10) and (11), we can decompose the MMPI into the following:

(12) MMPI=EC×BPC×PTCU×FCU

## 4. Data

### 4.1. City Group Classification

As the economic strength and the population density of the central cities increase, the central cities have a driving force effect on the surrounding area, which, in turn, forms a city circle; then, with the development of traffic conditions, such as highways, the boundaries of the urban circle have become blurred, and the adjacent urban circles have spread to form a city group with closer economic, demographic, environmental, and social relationships. China’s haze pollution has typical city group characteristics. As China’s urbanization accelerates, cities become increasingly dense, and the distance between cities becomes smaller and smaller, which is not conducive to the spread of atmospheric pollutants. Taking the city groups in south Jiangsu and central Liaoning as examples, it is said that there is almost no gap between cities. As a result, it is difficult for each city not only to purify its own pollutants but also to “separate” from each other. At the same time, the city groups reduce the amount of open space, and atmospheric circulation is hindered, making the pollutants stay in the cities longer. Therefore, China’s air pollution is no longer a problem for one city. Air pollution is becoming a common regional problem [54]. The mutual influence of air pollution between cities is becoming increasingly obvious, and the changes in air pollution between cities are also significantly synchronized [55].

This paper uses city panel data to analyse China’s environmental efficiency and technology gap under the influence of PM_2.5_ pollution. China’s top ten city groups basically cover the areas where most of China’s regional economic development is relatively high. The top ten city groups in China and the cities that they contain are defined as follows (see Table 1):

### 4.2. Data Sources

This paper uses labour (L) and physical capital (K) as the input variables, using the gross regional product (GRP) of each city as the desirable output variable and the PM_2.5_ concentration (b) as the undesired output variable. Except for the PM_2.5_ concentration data, the variables are mainly derived from the China City Statistical Yearbook (2004–2016):

Labour input (L). The labour input generally uses the full-time equivalent of the practitioner as a proxy variable. However, due to the lack of data on the working hours of labourers, the number of employees in each city is used.

Physical capital investment (K). Physical capital investment must be represented using the stock variable rather than the flow variable. Physical capital always uses the perpetual inventory method to transfer physical capital investment to physical capital stock [56,57]. Based on the data available, physical capital investment uses fixed asset investment as a proxy variable. Similar to Coe and Helpman [58] and Coe et al. [59], 5% is taken as the depreciation rate. Due to the lack of fixed asset investment price indexes in each city, we use the fixed asset price index of the corresponding provinces for each city.

Gross regional product (GRP). GRP is used as the output variable because the China City Statistical Yearbook reports the gross regional product only at the current price index. We use the GRP index of the corresponding provinces in each city to deflate the gross regional product to the 2004 constant price.

PM_2.5_ concentration data (PM_2.5_). Obtaining data on the PM_2.5_ concentration is a major difficulty in this paper. The Socioeconomic Data and Applications Center at Columbia University provides data on the PM_2.5_ average concentration based on satellite data using aerosol optical thickness (AOD) methods [60]. Ma et al. [61] used a two-stage approach based on ground data and satellite data, providing more accurate PM_2.5_ data. Therefore, our PM_2.5_ data are mainly derived from this method. However, Ma’s data are available only until 2013. The Information Centre of the Ministry of Environmental Protection of China provides official PM_2.5_ data after 2013, and the PM_2.5_ data after 2014 to 2016 come from the Environmental Protection Information Website of the Ministry of Environmental Protection.

Actual PM_2.5_ concentration data (Actual PM_2.5_). In addition, because PM_2.5_ has a strong spatial correlation, locally produced fine particulate contaminants are likely to spread to other regions through the effects of air and wind flow [55]; therefore, we must calculate whether PM_2.5_ is affected by outside sources of pollution in other regions [62]. Xue et al. [63] developed a transport matrix of PM_2.5_ and its chemical components from 31 provinces (sources) and 333 cities (receptors) by applying particulate source apportionment technology (PSAT) via the CAMx model. They obtained a PM_2.5_ mutual transmission matrix between 31 provinces and cities; finally, they obtained the contribution of external sources to PM_2.5_ and its chemical components for all provinces (see Table 2). We assigned these provincial-level data to the city level.

Finally, the descriptive statistics for each variable of China’s ten major city groups are shown in Table 3.

## 5. Results and Discussions

### 5.1. Environmental Efficiency of the EBM Model

The EBM model uses a hybrid distance function to estimate the efficiency of environmental technology. It combines the advantages of the SBM model and the radial model in the presence of undesired outputs so that more accurate estimates can be obtained from the EBM model. This section uses the EBM model to estimate environmental efficiency in the case of PM_2.5_ as an undesired output.

### 5.2. PM_2.5_ Environmental Efficiency

Environmental efficiency represents the public’s attitude towards air pollution, which requires reducing PM_2.5_ pollution, not excessively damaging economic growth, and achieving coordinated development between the environment and economic growth. Table 4 lists PM_2.5_ environmental efficiency with or without considering the impact of external sources and its ranking in major cities using Equation (6).

Table 4 shows that, on average, the overall annual environmental efficiency of PM_2.5_ is 0.525 without the external source impact and 0.531 with the external source impact. PM_2.5_ environmental efficiency is relatively low due to China’s extensive economic growth model. In addition, due to the spatial correlation of haze pollution, the actual PM_2.5_ in each city will be reduced after considering the influence of external sources, resulting in an increase in PM_2.5_ environmental efficiency.

Notably, there are also large differences in PM_2.5_ environmental efficiency between cities. Without the impact of outside sources, the cities with the highest PM_2.5_ environmental efficiency are Shenzhen, Guangzhou, and Shanghai, followed by Dongguan, Quanzhou, and Foshan. With the impact of outside sources, the cities with the highest PM_2.5_ environmental efficiency are Shanghai, Shenzhen, and Guangzhou, followed by Dongguan, Quanzhou, and Foshan. These cities with the highest environmental efficiency are all eastern cities. Among the four first-tier cities (Beijing, Shanghai, Guangzhou, Shenzhen) in China, only Beijing’s PM_2.5_ environmental efficiency is relatively low because Beijing is an inland city, unlike the other three cities, which are coastal cities. Geological conditions also determine that it is difficult for the PM_2.5_ pollution in Beijing to spread. Therefore, the PM_2.5_ pollution in Beijing is more serious than in the other three cities, which also leads to its relatively low environmental efficiency. Shanghai’s performance is particularly noteworthy; without the influence of outside sources, Shanghai ranks third, but with the influence of outside sources, it ranks first. This finding indicates that Shanghai’s haze pollution is more susceptible to outside pollution sources, and its PM_2.5_ environmental efficiency drops significantly when considering outside sources. In PM_2.5_ environmental efficiency, Hengshui, Xi’an, Huzhou, and Tongchuan have the lowest rankings. These cities are second- and third-tier cities in the central and western regions of China. The economic aggregates in these regions are not very large, and the economic structure is dominated by pollution-intensive industries. Therefore, the PM_2.5_ pollution in these cities is very serious, and the coordination between environmental pollution and economic growth is still insufficient; these cities face greater pressure to reduce emissions. The Chinese government still needs to boost its efforts to narrow the economic development between the central and western regions and the eastern region.

In addition, measuring PM_2.5_ environmental efficiency and the frontier cities based on the ten different city groups is very meaningful; the results are shown in Table 5.

The environmental efficiency frontier represents the most environmentally efficient cities compared to the other cities in the city group. These are also the cities with the most coordinated environmental pollution and economic growth. Table 5 shows that the frontiers of environmental efficiency in the group are generally the central cities of the city groups with relatively high economic development, such as provincial capitals. Under the administrative system of China, the central city not only has economic and natural resource-related advantages but can also transfer environmental pollution to peripheral cities through a series of administrative means, such as industrial transfer; as a result, the coordination of environmental and economic growth in central cities is higher than that in peripheral cities. Take Beijing as an example; although Beijing’s PM_2.5_ environmental efficiency under the meta-frontier is not very high, it is an environmental frontier city in the BTH city group. Notably, Xi’an ranked 97th under the meta-frontier, but in its group, it is an environmental frontier city. Environmental efficiency shows the greatest difference not only within the city group but also between city groups.

The TGR reflects the technological distance between the meta-frontier and the group frontier. The group frontier represents the optimal environmental efficiency that can be achieved in a certain city. The meta-frontier is the envelope curve of the group frontier, which represents the optimal environmental efficiency that can be achieved in all samples. The greater the TGR is, the farther the distance between the group frontier and the meta-frontier, indicating that the overall environmental efficiency of this area is relatively low and that the environmental regulation of this area needs to be increased.

As shown in Table 6, overall, the average TGR is 0.661 and 0.666 with or without considering the impact of outside sources, respectively. In addition, regardless of whether the impact of outside sources is considered, the technology gaps are relatively small. The city groups that consider outside sources with higher environmental efficiency than those that do not consider outside sources include the YRD, BTH, Shandong Peninsula, the west coast of the Taiwan Strait, the Central Plains, and the middle reaches of the Yangtze River. Specifically in the YRD city group, considering the impact of outside sources is closer to the environmental frontier. This finding indicates that the pollution of the YRD city group is more easily affected by outside sources.

There are also great gaps in the TGR between the ten major city groups. Among them, the PRD city group ranks first, followed by the west coast of the Taiwan Strait, which are both located in the eastern region. This finding means that these city groups are not experiencing excessive environmental pollution despite experiencing rapid economic growth. The Guanzhong city group and the Central Plains city group are ranked lower; these city groups are both located in the central and western regions. This finding indicates that the economic and environmental development of these areas has not been achieved harmoniously and that it is necessary to intensify efforts to carry out environmental remediation and pollution protection for these city groups.

**Figure 1:**
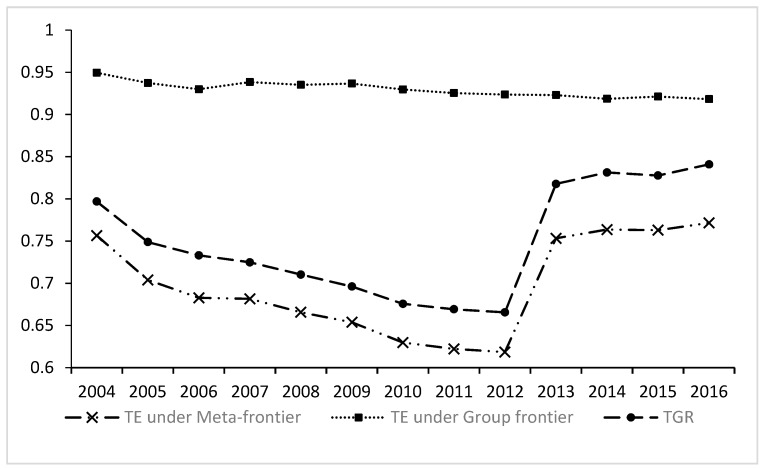
The dynamic trend of the annual average technology gap ratio and its decomposition.

Figure 1 shows the trend of the annual average TGR and its decomposition for annual average environmental efficiency under the meta-frontier and the group frontier. From the perspective of time trends, the TGR and environmental efficiency under the meta-frontier of the major city groups formed a turning point in 2012. Both the TGR and environmental efficiency declined slowly in 2004 to 2012 and gradually increased after 2013 to 2016. The PM_2.5_ problem in China has been the concern of public and government departments since 2012. Therefore, after 2012, the major city groups have begun to increase efforts to control air pollution. China has also introduced plans for air pollution prevention and control to reduce haze pollution. As a result, environmental efficiency began to increase gradually after 2012.

### 5.3. Analysis of the Meta-Frontier Malmquist Productivity Index

Equation (10) is used to measure the MMPI of each city group. The results are shown in Table 7 and Table 8.

Table 7 and Table 8 show that, overall, China’s environmental total factor productivity presents a significant downward trend. China’s overall environmental total factor productivity declined by 3.68% and 3.49% with and without the influence of outside sources, respectively. This conclusion fully shows that China’s economic growth over the past decade has come at the expense of air pollution. In addition, this conclusion is different from some studies in which the undesirable output is carbon emissions. Yao et al. [64] suggest that China’s environmental total factor productivity exhibits a slow upward trend. Fei and Lin [44] also believe that China’s agricultural productivity shows an upward trend. We speculate that there are two main reasons for the difference in results: On the one hand, there are differences in measurement methods. The measurement of carbon emissions is based on the production process, but the measurement of PM_2.5_ pollution is mainly based on satellite data. On the other hand, haze pollution is a more complex type of pollution that may be affected by many factors other than carbon emissions.

At the same time, from the perspective of city groups, we can find that the environmental efficiency changes between city groups also show great heterogeneity. However, the environmental efficiency of most city groups shows a downward trend. When considering external sources, only the PRD and Shandong Peninsula city groups showed an upward trend. When the external sources are not considered, only the YRD city group and the Shandong Peninsula city group show an upward trend. The total factor productivity of the other city groups has declined to varying degrees. In particular, some city groups, such as city group 8, show a significant decline in the MMPI. The declines were as high as 8.53% and 8.89% with or without the impact of outside sources, respectively. This finding further confirms our conclusion that most cities in China have not achieved coordinated development between the environment and economic growth. This conclusion is also very important for government decision makers. The MMPI shows the difficulty of improving environmental efficiency. We need to do more to control air pollution in cities with a lower MMPI.

From a dynamic perspective, we found that China’s overall MMPI showed a downward trend before 2013. In some years, such as 2004–2005 and 2010–2011, the decline trend is more obvious, and only after 2014 is an upward trend shown. From the perspective of city groups, most city groups have basically shown the same dynamic trends as those of city group 1, city group 2, and city group 5. This conclusion is roughly the same as that for the environmental efficiency trend.

### 5.4. Analysis of the Driving Factors of Meta-Frontier Malmquist Productivity Index

Furthermore, to analyse the causes of environmental efficiency changes, we decompose environmental efficiency utilizing Equation (12), and the measurement results are shown in Table 9.

(1) Efficiency change (EC) index. We find that there is also a large difference between the changes in the EC index. Overall, efficiency showed a large downward trend, and the overall efficiency was 0.9976, which means that efficiency decreased by 0.24%. This finding indicates that the catch-up process of the existing technological frontier is declining and that the environmental efficiency of each city group has not increased significantly. From the perspective of city groups, only the EC index of city groups 2, 3, and 4 showed a small increasing trend, with growth rates of 0.2%, 0.4%, and 0.8%, respectively. This finding means that there is still a great deal of space for catching up with the technology frontier.

**Figure 2:**
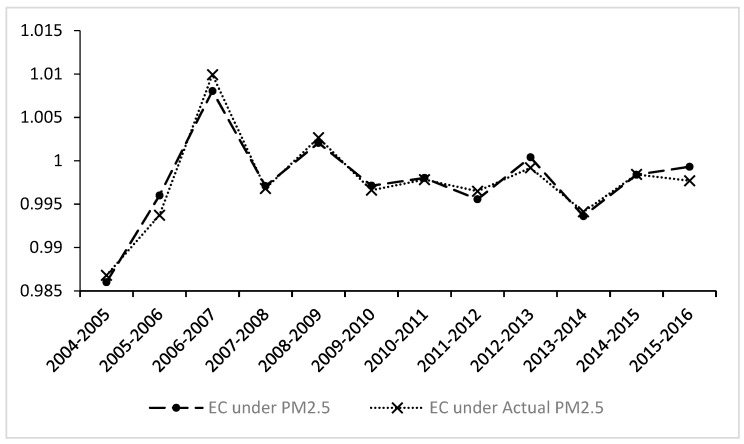
The dynamic trend of efficiency change (EC) index under PM_2.5_ (diameter of fine particulate matter less than 2.5 μm) and actual PM_2.5_.

In terms of the time trend, as shown in Figure 2, the EC index increased only by 0.80% in 2006–2007, increased by 0.21% in 2008–2009, and increased by 0.40% in 2012–2013. In other years, there was a significant decline, demonstrating that the chasing effect of the common technology frontiers in various groups in China is not ideal.

(2) Best practice gap change (BPC) index. The BPC index indicates the technology gap movement between the current technological frontier and the inter-temporal technological frontier. Overall, the BPC index also shows a downward trend; the BPC index is 0.9876 and 0.9873 with and without the impact of outside sources, respectively, which indicates that the environmental frontier is moving farther from the inter-temporal frontier at a decrease rate of 1.24%. This finding also shows that the technology of China’s major city groups is declining. From the perspective of city groups, only city group 1 and city group 5 have increased by 0.50% and 0.75%, respectively, which indicates that technological progress has been achieved. However, the technology progress of other city groups has not been significantly realized.

**Figure 3:**
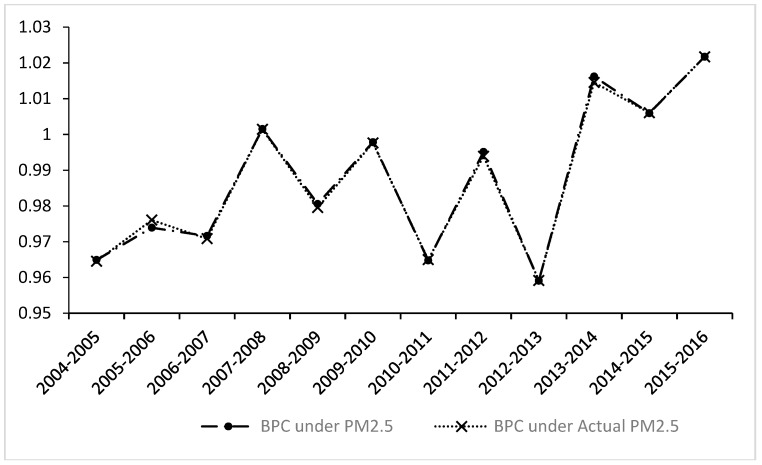
The dynamic trend of best practice gap change (BPC) index under PM_2.5_ and actual PM_2.5_.

From a dynamic trend perspective (see Figure 3), overall, similar to the MMPI and EC trends, the growth in the BPC index is roughly similar to that in the MMPI, but it fluctuates greatly. In addition to achieving a growth of 0.15% in 2007–2008, before 2013, a growth rate of only 0.15% was achieved in 2007–2008, but growth has also been achieved since 2013, indicating that the technological progress effect was mainly achieved after 2013.

(3) Pure technological catch-up index (PTCU). According to Equation (11), we can divide the TGC index into the PTCU and FCU indexes. The TGC index measures the technology gap between the global environment frontier and the inter-temporal environmental frontier. The PTCU index purely captures the catch-up in technology without the elements of technological inefficiency from the view of a group frontier. As seen from our measurement results, the PTCU index increased by 0.44% when considering external sources and increased by 0.40% when not considering external sources. This finding indicates that PTCU has a positive effect on the MMPI. Overall, this index is the only growth indicator among the four indicators. In regard to PTCU, the positive effect of the total factor productivity of the environment is more obvious.

**Figure 4:**
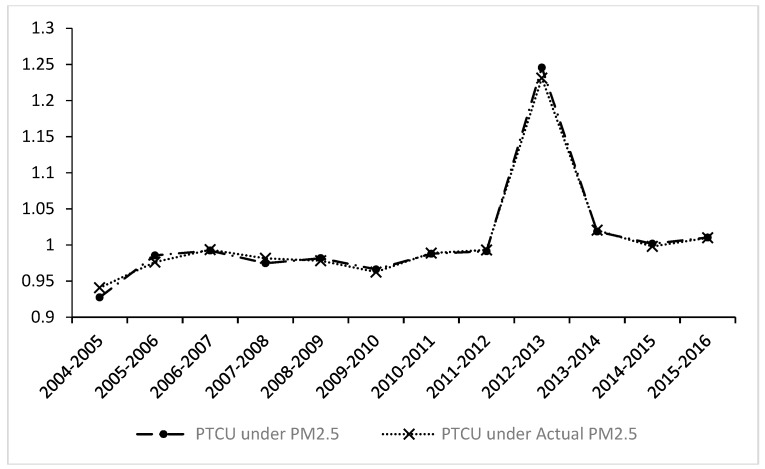
The dynamic trend of the pure technological catch-up (PTCU) index under PM_2.5_ and actual PM_2.5_.

From a dynamic perspective (see Figure 4), the trend of the PTCU index is consistent with the MMPI. The PTCU index showed a downward trend before 2012 and began to rise after 2012.

(4) Frontier catch-up (FCU). It is easier to determine if the FCU index captures the velocity of change of the meta-frontier relative to that of the group frontier. When the upward shift of the group frontier is faster than that of the meta-frontier, the FCU index will exhibit a value less than unity. Overall, with and without the impact of outside sources, the FCU index decreased by 2.47% and 2.53%, respectively. The downward trend of this indicator is the largest among the four indicators, demonstrating that the meta-frontier has a slower catch-up trend with respect to the group frontier and that the group frontier efficiency of some city groups is growing faster. Accordingly, we can observe that the decline in environmental total factor productivity is mainly due to the decline in FCU.

**Figure 5:**
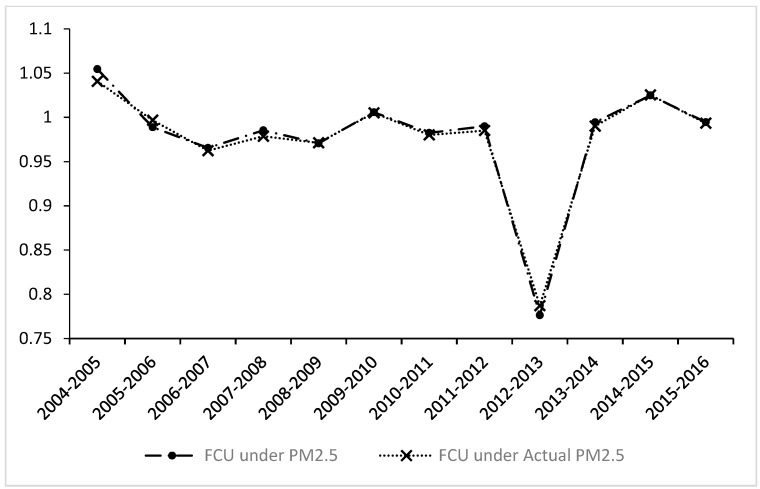
The dynamic trend of the frontier catch-up (FCU) index under PM_2.5_ and actual PM_2.5_.

As shown in Figure 5, unlike the other indexes, the FCU index shows a different trend; except for the rises in 2004–2005, 2009–2010, and 2014–2015, most years showed a downward trend.

### 5.5. Further Discussion

As have been mentioned before in Section 2, DEA has been employed in a number of studies to measure the performance of China’s environmental efficiency (Sueyoshi, Yuan, and Goto [8]). The meta-frontier approach has also been used in China to do efficiency measurement in many aspects while most of them incorporated the CO_2_ emissions as undesirable output (Yao, Guo, Shao, and Jiang [39], Fei and Lin [44], Zhang, Wang, and Chen [4]). However, very few works considered the PM_2.5_ pollution as an undesirable output. The most similar work to the current research is [47] on the measurement of efficiency of China’s provincial capitals in two annual periods (2013–2014) and [50] on the measurement the air quality of 31 main cities in China by using the daily Air Quality Index and integer and zero-sum gain constraints into DEA approach. However, [47] only used the rank sum test to find the difference in city groups, [50]’s study did not consider the difference in city groups and our study uses meta-frontier Malmquist productivity index to analyze their difference. But all studies also found the efficiency in four municipalities is higher than in other cities. Furthermore, in our study unlike [47], we use PM_2.5_ data from remote sensing data (AOD) and the official department, while [47] used the simulation data and [50] only used the official data. This study is the first study using a meta-frontier Malmquist productivity approach incorporating PM_2.5_ as an undesirable outputs using a new epsilon-based DEA model to provide more accurate measures of environmental efficiency and productivity change. And the study in [50] measured the air quality of 31 main cities in China by using the daily Air Quality Index.

## 6. Conclusions

This paper first uses the EBM model to measure the environmental efficiency of China’s top ten city groups under the meta-frontier and the group frontier and then uses the meta-frontier model to measure the total factor productivity and to decompose it. The measurement results find the overall annual environmental efficiency of PM_2.5_ is 0.525 without the external source impact and 0.531 with the external source impact and indicate that there is a large gap in environmental efficiency among these ten city groups. The gap between the eastern regions and the western regions is still obvious. The cities with the highest environmental efficiency rankings are generally the cities along the eastern coast. The environmental efficiencies of Shanghai, Shenzhen, and Guangzhou are among the top three cities. In addition, the environmental group frontiers are mainly central cities and economically developed cities.

In addition, China’s overall environmental total factor productivity declined by 3.68% and 3.49% with and without the influence of outside sources, respectively. From a dynamic perspective, China’s overall MMPI index showed a downward trend before 2013. Only a few eastern coastal city groups have increased their environmental efficiency. This downward trend shows that China’s environmental pollution is still relatively serious. China has not achieved coordinated development between the environment and economic growth in the past decade. Furthermore, China has not developed a good environmental protection system during this time. This conclusion has important practical significance for China, which emphasizes economic transformation and high-quality economic development. China needs to intensify its efforts to improve the quality of development, especially to increase control over environmental pollution, and it must use environmental regulations to improve environmental efficiency.

The overall efficiency of EC, BPC, PTCU, FCU are 0.9976, 0.9876, 1.0044, 0.9753, respectively, without the external source impact and 0.9975, 0.9873, 1.0040, 0.9742, respectively, with the external source impact. The decomposition of environmental total factor productivity shows that the decline in China’s environmental total factor productivity is mainly caused by FCU. At the same time, EC and BPC also have a significant negative effect on environmental total factor productivity. This finding shows that the technology gap between the groups is still relatively obvious, which fully proves that the development between China’s city groups remains uncoordinated and insufficient.

From the conclusions of this paper, some measures should be adopted to achieve coordinated development between the environment and the economy. First, it is necessary to increase efforts to improve environmental efficiency. Improving environmental efficiency can accelerate economic growth while avoiding damage to economic growth and represents an effective means of harmonious growth in China. Second, it is necessary to adopt some measures to overcome the imbalance between the city groups and within the city groups. For example, China can transfer advanced environmental technologies and advanced environmental measures from the eastern city groups to the central and western city groups. Third, because there are great efficiency gaps and technological progress gaps between city groups, China needs to accelerate the narrowing of the technological efficiency gap and technological progress gap between city groups. As our measurement results show, there is still much room for technological improvement in China. China can design some mechanisms, such as environmental taxation and energy quota trading systems, to narrow the gap.

## Figures and Tables

**Table 1:** The city group classification of the ten major city groups in China.

City Group	Group ID	Cities Included in the City Group
Yangtze River Delta	1	Zhenjiang, Taizhou (in Jiangsu), Hangzhou, Huzhou, Shaoxing, Suzhou, Hefei, Taizhou (in Zhejiang), Changzhou, Nantong, Wuxi, Jiaxing, Yangzhou, Yancheng, Shanghai Jinhua, Nanjing, Zhoushan, Ningbo
Pearl River Delta	2	Zhaoqing, Jiangmen, Shenzhen Huizhou, Dongguan, Zhongshan, Zhuhai, Foshan
Beijing-Tianjin-Hebei	3	Cangzhou, Handan, Beijing, Langfang, Tianjin, Shijiazhuang, Zhangjiakou, Xingtai, Tangshan, Chengde, Hengshui, Baoding, Qinhuangdao
Central and southern Liaoning	4	Benxi, Tieling, Shenyang, Liaoyang, Panjin, Dandong, Dalian Fushun, Anshan, Yingkou
Shandong Peninsula	5	Weihai, Jinan, Dongying, Qingdao Zibo, Rizhao, Weifang, Yantai
Cheng Yu	6	Chengdu, Deyang, Chongqing, Mianyang, Neijiang, Zigong, bSuining, Luzhou
West coast of the Taiwan Strait	7	Zhangzhou, Ningde, Putian, Xiamen, Quanzhou, Fuzhou
Central Henan	8	Pingdingshan, Xinxiang, Jiaozuo, Luohe, Zhengzhou, Xuchang, Kaifeng, Luoyang
Middle reaches of the Yangtze River	9	Ezhou, Suizhou, Yueyang, Jingmen, Huanggang, Jingzhou, Huangshi, Xianning, Wuhan, Xinyang, Jiujiang, Xiaogan
Guanzhong	10	Weinan, Shangluo, Xianyang, Tongchuan, Baoji, Xi’an

**Table 2:** Contribution of outside sources to PM_2.5_ (diameter of fine particulate matter less than 2.5 μm) (%).

Province	Contribution	Province	Contribution
Beijing	37	Hubei	42
Tianjin	42	Hunan	39
Hebei	36	Guangdong	35
Shanxi	31	Guangxi	46
Inner Mongolia	22	Hainan	71
Liaoning	33	Chongqing	31
Jilin	48	Sichuan	28
Heilongjiang	20	Guizhou	37
Shanghai	54	Yunnan	36
Jiangsu	50	Tibet	1
Zhejiang	48	Shaanxi	31
Anhui	42	Gansu	33
Fujian	41	Qinghai	13
Jiangxi	48	Ningxia	35
Shandong	41	Xinjiang	0
Henan	37	National Mean	36

**Table 3:** Descriptive statistics for each variable of China’s ten major city groups.

Variable	Unit	Obs	Mean	Std. Dev.	Min	Max
GRP	Billion yuan	1287	2254.01	2782.91	58.90	23,423.39
Capital stock	Billion yuan	1287	741,092.30	896,380.40	6078.33	6,892,826.00
Labour	10 thousand persons	1287	83.58	112.85	9.07	986.87
PM_2.5_ Concentration	μg/m^3^	1287	68.89	21.39	23.14	125.33
Actual PM_2.5_ Concentration	μg/m^3^	1287	42.24	14.17	12.83	80.22

**Table 4:** The PM_2.5_ environmental efficiency and its ranking under meta-frontier during 2004 to 2016.

DMU	PM_2.5_	Rank	Actual PM_2.5_	Rank	DMU	PM_2.5_	Rank	Actual PM_2.5_	Rank
Anshan	0.625	18	0.624	21	Qinhuangdao	0.498	53	0.498	55
Baoji	0.435	80	0.434	82	Qingdao	0.712	13	0.727	13
Baoding	0.371	95	0.372	95	Quanzhou	0.872	5	0.889	5
Beijing	0.685	14	0.670	15	Rizhao	0.533	40	0.534	42
Benxi	0.435	81	0.435	81	Shangluo	0.402	88	0.401	90
Cangzhou	0.657	15	0.658	16	Shanghai	0.964	3	1.000	1
Changzhou	0.553	34	0.589	26	Shaoxing	0.463	72	0.497	57
Chengdu	0.486	62	0.467	72	Shenzhen	0.991	1	0.984	2
Chengde	0.469	68	0.470	70	Shenyang	0.570	29	0.562	34
Dalian	0.747	10	0.730	12	Shijiazhuang	0.489	60	0.490	62
Dandong	0.492	56	0.492	60	Suzhou	0.790	8	0.852	7
Deyang	0.586	27	0.585	28	Suizhou	0.551	35	0.553	37
Dongguan	0.906	4	0.906	4	Suining	0.464	71	0.464	74
Dongying	0.577	28	0.580	29	Xiamen	0.541	39	0.552	38
Ezhou	0.465	70	0.466	73	Taizhou (in Zhejiang)	0.506	50	0.550	41
Foshan	0.871	6	0.871	6	Taizhou (in Jiangsu)	0.480	64	0.488	64
Fuzhou	0.615	21	0.633	19	Tangshan	0.783	9	0.787	10
Fushun	0.525	42	0.525	45	Tianjin	0.728	12	0.755	11
Guangzhou	0.966	2	0.958	3	Tieling	0.443	77	0.443	78
Handan	0.512	49	0.513	51	Tongchuan	0.369	96	0.369	96
Hangzhou	0.619	20	0.645	18	Weihai	0.459	75	0.473	69
Hefei	0.445	76	0.450	77	Weifang	0.441	78	0.457	76
Hengshui	0.254	99	0.254	99	Weinan	0.428	83	0.428	84
Huzhou	0.350	98	0.360	97	Wuxi	0.729	11	0.816	8
Huanggang	0.395	90	0.397	92	Wuhan	0.554	33	0.577	30
Huangshi	0.490	58	0.491	61	Xi’an	0.357	97	0.351	98
Huizhou	0.498	54	0.498	56	Xianning	0.436	79	0.437	79
Jinan	0.514	48	0.519	46	Xianyang	0.403	87	0.402	89
Jiaxing	0.386	93	0.418	86	Xiaogan	0.410	86	0.411	88
Jiangmen	0.594	25	0.594	25	Xinxiang	0.375	94	0.375	94
Jiaozuo	0.463	73	0.463	75	Xinyang	0.391	92	0.391	93
Jinhua	0.393	91	0.432	83	Xingtai	0.420	84	0.421	85
Jingmen	0.491	57	0.493	58	Xuchang	0.551	36	0.552	39
Jingzhou	0.478	66	0.479	67	Yantai	0.598	23	0.614	22
Jiujiang	0.431	82	0.435	80	Yancheng	0.489	59	0.501	53
Kaifeng	0.566	31	0.567	33	Yangzhou	0.506	51	0.515	50
Langfang	0.481	63	0.481	65	Yingkou	0.478	67	0.477	68
Liaoyang	0.595	24	0.595	24	Yueyang	0.623	19	0.625	20
Luzhou	0.493	55	0.492	59	Zhangjiakou	0.480	65	0.480	66
Luoyang	0.524	43	0.525	44	Zhangzhou	0.646	17	0.654	17
Luohe	0.517	47	0.517	49	Zhaoqing	0.551	37	0.551	40
Mianyang	0.501	52	0.499	54	Zhenjiang	0.542	38	0.554	36
Neijiang	0.587	26	0.586	27	Zhengzhou	0.487	61	0.489	63
Nanjing	0.523	44	0.571	32	Zhongshan	0.529	41	0.529	43
Nantong	0.466	69	0.518	48	Chongqing	0.520	45	0.502	52
Ningbo	0.647	16	0.677	14	Zhoushan	0.415	85	0.418	87
Ningde	0.569	30	0.571	31	Zhuhai	0.517	46	0.518	47
Panjin	0.397	89	0.397	91	Zibo	0.461	74	0.467	71
Pingdingshan	0.558	32	0.559	35	Zigong	0.792	7	0.791	9
Putian	0.603	22	0.607	23	Mean	0.525	---	0.531	---

**Table 5:** The PM_2.5_ environmental efficiency frontier cities based on the group-frontier during 2004 to 2016.

City Group	Group ID	City
Yangtze River Delta	1	Shanghai
Pearl River Delta	2	Shenzhen
Beijing-Tianjin-Hebei	3	Beijing, Tangshan, Tianjin
Central and southern Liaoning	4	Dalian
Shandong Peninsula	5	Dongying, Jinan, Qingdao
Cheng Yu	6	Chengdu, Deyang, Chongqing, Zigong
West Coast of Taiwan Straits	7	Quanzhou, Zhangzhou
Central Henan	8	Kaifeng, Luoyang, Xuchang, Zhengzhou
Middle reaches of the Yangtze River	9	Wuhan, Yueyang
Guanzhong	10	Baoji, Xi’an

**Table 6:** Technology gap ratio under PM_2.5_.

City Group	Group ID	TGR (PM_2.5_)	TGR (Actual PM_2.5_)
Yangtze River Delta	1	0.695	0.735
Pearl River Delta	2	0.990	0.988
Beijing-Tianjin-Hebei	3	0.712	0.713
Central and southern Liaoning	4	0.692	0.689
Shandong Peninsula	5	0.588	0.598
Cheng Yu	6	0.599	0.593
West coast of Taiwan Strait	7	0.699	0.709
Central Henan	8	0.559	0.560
Middle reaches of the Yangtze River	9	0.646	0.647
Guanzhong	10	0.434	0.432
Mean		0.661	0.666

**Table 7:** Changes in the meta-frontier Malmquist total factor productivity index (MMPI) in ten major city groups under PM_2.5_ constrictions.

	Group ID	Group 1	Group 2	Group 3	Group 4	Group 5	Group 6	Group 7	Group 8	Group 9	Group 10	Mean
Year												
2004–2005		0.9890	1.0296	0.9372	0.9390	0.9749	0.9201	0.8975	0.8552	0.8495	0.9282	0.9305
2005–2006		1.0198	1.0034	0.9431	0.8658	1.0138	0.9587	0.9974	0.8582	0.9123	0.8983	0.9453
2006–2007		0.9522	1.0076	0.9138	0.9097	1.0466	0.9619	0.9184	0.8677	0.9006	0.9182	0.9383
2007–2008		0.9819	1.0243	0.9644	0.9185	1.0306	0.9401	0.9768	0.9143	0.9313	0.9165	0.9590
2008–2009		1.0246	1.0142	0.9042	0.8732	0.9719	0.8745	1.0441	0.8550	0.8919	0.9328	0.9363
2009–2010		1.0170	1.0918	0.9608	0.9824	1.0113	0.9345	1.0124	0.8918	0.8863	0.9009	0.9669
2010–2011		0.9776	0.9326	0.9380	0.9323	0.9794	0.9490	0.9263	0.9274	0.8992	0.8862	0.9344
2011–2012		0.9947	1.0243	1.0125	0.9506	1.0173	0.9445	0.9566	0.9180	0.9391	0.9709	0.9722
2012–2013		0.9133	0.8813	0.9941	0.9777	0.9785	0.8755	0.9585	0.8938	0.9293	0.8858	0.9278
2013–2014		1.0288	1.0121	1.0319	0.9723	1.1102	1.0662	0.9802	1.0137	0.9744	1.0453	1.0227
2014–2015		1.0479	1.0436	1.0482	1.0394	1.0641	0.9936	1.0612	0.9941	0.9993	1.0220	1.0310
2015–2016		1.0588	0.9869	1.0549	1.0575	1.0370	1.0113	1.0434	1.0070	1.0310	0.9754	1.0259
Mean		0.9997	1.0030	0.9740	0.9499	1.0189	0.9511	0.9798	0.9147	0.9274	0.9387	0.9651

**Table 8:** Changes in the MMPI in ten major city groups under actual PM_2.5_ constrictions.

	Group ID	Group ID	Group 2	Group 3	Group 4	Group 5	Group 6	Group 7	Group 8	Group 9	Group 10	Mean
Year												
Year			1.0364	0.9416	0.9328	0.9691	0.9174	0.9074	0.8529	0.8547	0.9279	0.9318
2005–2006		1.0216	1.0082	0.9480	0.8724	0.9989	0.9566	0.9926	0.8568	0.9128	0.8881	0.9439
2006–2007		0.9514	1.0124	0.9166	0.9134	1.0466	0.9612	0.9208	0.8535	0.9034	0.9090	0.9373
2007–2008		0.9848	1.0219	0.9635	0.9128	1.0362	0.9378	0.9827	0.9118	0.9331	0.9135	0.9588
2008–2009		1.0251	1.0070	0.9024	0.8701	0.9672	0.8738	1.0371	0.8529	0.8891	0.9286	0.9331
2009–2010		1.0292	1.0621	0.9572	0.9687	1.0073	0.9275	1.0082	0.8911	0.8876	0.8977	0.9619
2010–2011		0.9748	0.9406	0.9390	0.9219	0.9795	0.9487	0.9302	0.9259	0.8918	0.8852	0.9333
2011–2012		0.9883	1.0154	1.0147	0.9418	1.0219	0.9325	0.9612	0.9177	0.9436	0.9578	0.9688
2012–2013		0.9289	0.8804	0.9842	0.9593	0.9829	0.8717	0.9763	0.8922	0.9316	0.8887	0.9287
2013–2014		1.0299	1.0126	1.0362	0.9740	1.1077	1.0533	0.9889	1.0095	0.9669	1.0186	1.0190
2014–2015		1.0594	1.0294	1.0412	1.0402	1.0566	0.9802	1.0731	0.9886	0.9889	1.0251	1.0278
2015–2016		1.0706	0.9710	1.0488	1.0568	1.0434	1.0167	1.0289	0.9999	1.0199	0.9744	1.0225
Mean		1.0039	0.9987	0.9733	0.9454	1.0173	0.9468	0.9828	0.9111	0.9259	0.9334	0.9632

**Table 9:** The decomposition of MMPI in each city group.

Group ID	PM_2.5_				Actual PM_2.5_			
	EC (PM_2.5_)	BPC (PM_2.5_)	PTCU (PM_2.5_)	FCU (PM_2.5_)	EC (APM_2.5_)	BPC (APM_2.5_)	PTCU (APM_2.5_)	FCU (APM_2.5_)
Group 1	0.9930	1.0050	1.0100	0.9918	0.9929	1.0043	1.0068	1.0000
Group 2	1.0004	0.9905	1.0070	1.0051	1.0004	0.9905	1.0075	1.0003
Group 3	1.0004	0.9835	1.0096	0.9806	1.0004	0.9834	1.0101	0.9794
Group 4	1.0008	0.9778	0.9961	0.9745	1.0008	0.9778	0.9963	0.9698
Group 5	0.9988	1.0075	1.0227	0.9901	0.9988	1.0075	1.0215	0.9897
Group 6	0.9986	0.9913	1.0035	0.9575	0.9986	0.9911	1.0040	0.9529
Group 7	0.9955	0.9988	0.9980	0.9873	0.9955	0.9988	0.9951	0.9933
Group 8	0.9983	0.9723	0.9883	0.9536	0.9983	0.9723	0.9884	0.9496
Group 9	0.9932	0.9665	1.0068	0.9597	0.9921	0.9652	1.0075	0.9596
Group 10	0.9973	0.9834	1.0025	0.9548	0.9973	0.9834	1.0026	0.9493
Mean	0.9976	0.9876	1.0044	0.9753	0.9975	0.9873	1.0040	0.9742
